# A high-throughput and multiplex microsphere immunoassay based on non-structural protein 1 can discriminate three flavivirus infections

**DOI:** 10.1371/journal.pntd.0007649

**Published:** 2019-08-23

**Authors:** Jasmine Tyson, Wen-Yang Tsai, Jih-Jin Tsai, Ludvig Mässgård, Susan L. Stramer, Axel T. Lehrer, Vivek R. Nerurkar, Wei-Kung Wang

**Affiliations:** 1 Department of Tropical Medicine, Medical Microbiology and Pharmacology, John A. Burns School of Medicine, University of Hawaii at Manoa, Honolulu, Hawaii, United States of America; 2 Tropical Medicine Center, Kaohsiung Medical University Hospital, Kaohsiung, Taiwan; 3 Division of Infectious Diseases, Department of Internal Medicine, Kaohsiung Medical University Hospital, Kaohsiung, Taiwan; 4 Center for Dengue Fever Control and Research, Kaohsiung Medical University, Kaohsiung, Taiwan; 5 School of Medicine, College of Medicine, Kaohsiung Medical University, Kaohsiung, Taiwan; 6 Faculty of Medicine and Health Sciences, Linköping University, Linköping, Sweden; 7 American Red Cross Scientific Support Office, Gaithersburg, Maryland, United States of America; Duke-NUS GMS, SINGAPORE

## Abstract

The explosive spread of Zika virus (ZIKV) and associated complications in flavivirus-endemic regions underscore the need for sensitive and specific serodiagnostic tests to distinguish ZIKV, dengue virus (DENV) and other flavivirus infections. Compared with traditional envelope protein-based assays, several nonstructural protein 1 (NS1)-based assays showed improved specificity, however, none can detect and discriminate three flaviviruses in a single assay. Moreover, secondary DENV infection and ZIKV infection with previous DENV infection, both common in endemic regions, cannot be discriminated. In this study, we developed a high-throughput and multiplex IgG microsphere immunoassay (MIA) using the NS1 proteins of DENV1-DENV4, ZIKV and West Nile virus (WNV) to test samples from reverse-transcription-polymerase-chain reaction-confirmed cases, including primary DENV1, DENV2, DENV3, WNV and ZIKV infections, secondary DENV infection, and ZIKV infection with previous DENV infection. Combination of four DENV NS1 IgG MIAs revealed a sensitivity of 94.3% and specificity of 97.2% to detect DENV infection. The ZIKV and WNV NS1 IgG MIAs had a sensitivity/specificity of 100%/87.9% and 86.1%/78.4%, respectively. A positive correlation was found between the readouts of enzyme-linked immunosorbent assay and MIA for different NS1 tested. Based on the ratio of relative median fluorescence intensity of ZIKV NS1 to DENV1 NS1, the IgG MIA can distinguish ZIKV infection with previous DENV infection and secondary DENV infection with a sensitivity of 88.9–90.0% and specificity of 91.7–100.0%. The multiplex and high-throughput assay could be applied to serodiagnosis and serosurveillance of DENV, ZIKV and WNV infections in endemic regions.

## Author summary

Although there was a decrease of Zika virus (ZIKV) infection since late 2017, the specter of congenital Zika syndrome and its re-emergence in flavivirus-endemic regions emphasize the need for sensitive and specific serological tests to distinguish ZIKV, dengue virus (DENV) and other flaviviruses. Compared with traditional tests based on envelope protein, several nonstructural protein 1 (NS1)-based assays had improved specificity, however, none can discriminate three flaviviruses in a single assay. Moreover, secondary DENV infection and ZIKV infection with previous DENV infection, both common in endemic regions, cannot be distinguished. Herein we developed a high-throughput and multiplex IgG microsphere immunoassay using the NS1 proteins of four DENV serotypes, ZIKV and West Nile virus to test samples from laboratory-confirmed cases with different primary and secondary flavivirus infections. Combination of four DENV NS1 assays revealed a sensitivity of 94.3% and specificity of 97.2%. The ZIKV and WNV NS1 assays had a sensitivity/specificity of 100%/87.9% and 86.1%/78.4%, respectively. Based on the signal ratio of ZIKV NS1 to DENV1 NS1, the assay can distinguish ZIKV infection with previous DENV infection and secondary DENV infection with a sensitivity of 88.9–90.0% and specificity of 91.7–100.0%. This has applications to serodiagnosis and serosurveillance in endemic regions.

## Introduction

Despite a marked decrease of Zika virus (ZIKV) infection since late 2017, the specter of congenital Zika syndrome (CZS) and its re-emergence in flavivirus-endemic regions highlight the need for sensitive and specific diagnostic tests [1–4]. Similar to the laboratory diagnosis for other flaviviruses, detection of nucleic acid as soon as possible post-symptom onset (PSO) is considered as the gold standard to confirm ZIKV infection, [5,6]. Since many individuals test for ZIKV infection beyond the period when RNA is detectable and most (~80%) of ZIKV infections are asymptomatic, serological tests remain as a key component of ZIKV confirmation [5,6]. Furthermore, ZIKV can be transmitted sexually or following asymptomatic infection [7–9].

ZIKV is a member of the genus *Flavivirus* of the family *Flaviviridae*, which includes several pathogenic mosquito-borne viruses in different serocomplexes. The four serotypes of dengue virus (DENV) belong to the DENV serocomplex; West Nile virus (WNV) and Japanese encephalitis virus (JEV) to the JEV serocomplex; yellow fever virus (YFV) as a single member; and ZIKV^10^. Given that the envelope (E) protein is the major target of antibody response after flavivirus infection, different E antigens such as recombinant E protein, inactivated virions or virus-like particles have been developed for serological tests [10–13]. Due to the presence of several highly conserved residues of flavivirus E proteins, anti-E antibodies in serum are commonly cross-reactive to different flaviviruses [13–17]. The guidelines of Centers for Disease Control and Prevention (CDC) recommend that positive or equivocal results of E protein-based IgM tests require further testing with time-consuming plaque reduction neutralization tests (PRNT) [5,6]. However, PRNT can confirm ZIKV-infected individuals who acquire ZIKV as the first flavivirus infection, known as primary ZIKV (pZIKV) infection, but often can only be interpreted as unspecified flavivirus infections for those who have experienced previous DENV or other flavivirus infections, limiting its application for ZIKV serodiagnosis in flavivirus-endemic regions.

When 795 sera that were IgM positive for ZIKV antigen by ELISA were tested for flavivirus neutralizing antibodies by PRNT, 45% were positive for ZIKV and at least one other flavivirus [18]. This non-specificity may be an inherent property of the early post-infection response to ZIKV or reflect prior flavivirus experience. A large number of Americans (7 million) have experienced a WNV infection since 1999 [19] and ~8 million traveled to yellow fever endemic countries in 2015 [20,21]. Thus, a sensitive, specific and multiplex serological test that can distinguish ZIKV and other flavivirus infections is needed in both U.S. and flavivirus-endemic countries [18]. Moreover, several studies have shown that anti-DENV or WNV antibodies can enhance ZIKV infection *in vitro* [22–26] and in small animals, in which administration of DENV-immune plasma resulted in increased viremia and mortality in stat2 knock out mice [27]. This is known as antibody-dependent enhancement, in which antibody at suboptimal concentration for neutralization can enhance DENV, ZIKV or other flavivirus entry and replication in Fcγ receptor-bearing cells such as monocytes and is believed to contribute to disease pathogenesis [28]. Despite ADE of ZIKV by previous DENV immunity was not supported by two studies in non-human primates [29,30], more in-depth studies of DENV immunity on ZIKV disease outcome and complication in humans are warranted [31–33]. Thus, serological tests that can distinguish pZIKV infection (p = primary) from ZIKV infection with previous DENV (ZIKVwprDENV, wpr = with previous) infection are crucial to understand the pathogenesis of ZIKV and CZS in regions where ZIKV and DENV co-circulate.

Compared with traditional E protein-based assays, several enzyme-linked immunosorbent assays (ELISAs) based on ZIKV nonstructural protein 1 (NS1), including a recently reported blockade of binding ELISA, have shown improved specificity [34–39]. However, secondary DENV (sDENV) and ZIKVwprDENV infections, of which both were common in endemic regions, cannot be discriminated [34–39]. Moreover, none can detect and distinguish ZIKV, DENV and other flavivirus in a single assay.

With its high-throughput and multiplex (up to 100-plex) capacity, microsphere immunoassay (MIA) has been employed in the detection of cytokines, transplantation and transfusion antigens, and various bacterial and viral pathogens [40–43]. Previously, we reported that a combination of ELISAs based on the NS1 proteins of DENV and ZIKV can distinguish various DENV and ZIKV infections [44,45]. In this study, we developed a high-throughput and multiplex IgG MIA using NS1 proteins of DENV1 to DENV4, ZIKV and WNV, and showed that the NS1 IgG MIA can detect and distinguish not only primary DENV, ZIKV and WNV infections but also sDENV and ZIKVwprDENV infections.

## Methods

### Ethics statement and human samples

The Institutional Review Boards (IRB) of the University of Hawaii approved this study (CHS #17568, CHS#23786). S1 Table summarizes the numbers, serotypes, sampling time and sources of different panels of serum or plasma samples, including those from primary DENV1 (pDENV1), primary DENV2 (pDENV2), primary DENV3 (pDENV3), primary WNV (pWNV), pZIKV, sDENV and ZIKVwprDENV infections as well as flavivirus-naïve individuals. Samples collected <3 months or ≥3 months PSO were designated as convalescent- or post-convalescent-phase samples, respectively. Samples from reverse transcription-PCR (RT-PCR) confirmed Zika cases were from the Pediatric Dengue Cohort Study (PDCS) and the Pediatric Dengue Hospital-based Study in Managua, Nicaragua between July 2016 and March 2017 [46,47]. The Zika cases that were DENV-naïve or previously DENV-exposed were defined as pZIKV (p = primary) or ZIKVwprDENV (wpr = with previous) panels, respectively. The DENV-immune status was based on anti-DENV antibody testing by an inhibition ELISA at entry and annually of the PDCS [44–47]. Parents or legal guardians of all participants provided written informed consents, and participants ≥6-year old provided assents. These studies were approved by the IRBs of the University of California, Berkeley, and Nicaraguan Ministry of Health. Thirty-six plasma samples from blood donors, who were tested WNV-positive by the transcription-mediated amplification (a sensitive nucleic acid detection method used in blood bank), IgM and IgG antibodies between 2006 and 2015, designated as pWNV infection, were provided by the American Red Cross at Gaithersburg, Maryland [48]. Pre-2015-16 ZIKV epidemic convalescent- and post-convalescent-phase samples from RT-PCR confirmed cases with different primary DENV infections (pDENV1, pDENV2, and pDENV3) or sDENV infection were from Taiwan, Hawaii and Nicaragua; 53 flavivirus-naïve samples from a seroprevalence study in Taiwan were included as control in this study [44,45,49–52]. Samples from cases with primary DENV4 infection were not available. Primary DENV or sDENV infection was determined by IgM/IgG ratio or focus-reduction neutralization tests as described previously [49–51].

### Recombinant NS1 proteins

The NS1 gene (corresponding to amino acid residues 1–352) of ZIKV (HPF2013 strain) with a His-tag at the C-terminus was codon-optimized (Integrated DNA Technologies, Skokie, IL) and cloned into pMT-Bip vector to establish a Drosophila S2-cell stable clone [44]. ZIKV-NS1 protein from supernatants of the stable clone was purified by fast purification chromatography system (AKTA Pure, GE Health Care Bio-Science, Pittsburg, PA) [44]. Purified DENV1-4 and WNV NS1 proteins were purchased from The Native Antigen (Oxford, UK).

### Coupling of microspheres

Ten μg each of the 6 purified NS1 proteins, bovine serum albumin (BSA) and PBS (as negative antigen control) were coupled individually onto 8 types of magnetic carboxylated miscrosphere beads (1.25 X 10^6^ each) containing different fluorophores (MagPlexTM-C) (Luminex, TX, Austin) using two-step carbodiimide process at room temperature [53,54]. The antigen-conjugated microspheres were stored in 250 uL PBN buffer (PBS with 1% BSA and 0.05% sodium azide, Sigma Aldrich) at 4°C until use.

### MIA

Eight types of microsphere beads coupled with different NS1 proteins, BSA or PBS were combined and diluted in PBS-1% BSA. Fifty μL of the mixture (containing ~1250 beads of each type) were added to each well of a flat-bottom 96-well plate, and incubated with 50 μL diluted serum or plasma (1:100 dilution in PBS-1% BSA) at 37°C for 30 min in the dark, followed by wash with 200 μL of PBS-1% BSA twice, incubation with 50 μL of red phycoerythrin-conjugated anti-human or anti-mouse IgG (Jackson Immune Research Laboratory, West Grove, PA) at 37°C for 45 min in the dark, and wash with 200 μl of PBS-1% BSA twice [54]. Microspheres were then resuspended in 100 μl of PBS-1% BSA, incubated for 5 min and read by Luminex 200 machine (Austin, TX). All incubations were performed on a plate shaker at 700 rpm and all wash steps used a 96-well magnetic plate separator (Millipore Corp., Billerica, MA) [54]. Each plate includes two positive controls (confirmed-ZIKV or DENV infection), four negative controls (flavivirus-naïve samples), samples, and mouse anti-His mAb (all in duplicates). The median fluorescence intensity (MFI) was determined for 100 microspheres for each well. The MFI values for each antigen were divided by the mean MFI value of one positive control (MFI~10^4^) and multiplied by 10^4^ to calculate to rMFI for comparison between plates (S1 Fig). The cutoff rMFI for each antigen was defined by the mean rMFI value of 19 flavivirus-naïve samples plus 5 standard deviations, which gave a confidence level higher than 99.9% from 4 negatives [55]. Each MIA was performed twice (each in duplicate). New batch of conjugated antigens was tested with flavivirus-naïve panel to determine the cutoff rMFI.

### ELISAs

DENV1-, DENV2-, DENV3-, and ZIKV-NS1 IgG ELISAs have been described previously [44,45]. Briefly, purified NS1 proteins (16 ng for individual NS1 protein per well) were coated on 96-well plates at 4°C overnight, followed by blocking (StartingBlock blocking buffer, Thermo Scientific, Waltham, MA), incubation with primary antibody (serum or plasma at 1:400 dilution) and secondary antibody (anti-human IgG conjugated with horseradish peroxidase, Jackson Immune Research Laboratory, West Grove, PA), and wash [44,45]. After adding tetramethylbenzidine substrate (Thermo Scientific, Waltham, MA) followed by stop solution, the optical density (OD) at 450 nm was read with a reference wavelength of 630 nm. Each ELISA plate included two positive controls (confirmed-ZIKV or DENV infection), four negative controls (flavivirus-naïve sample), and samples (all in duplicate). The OD values were divided by the mean OD value of one positive control (OD close to 1) in the same plate to calculate the relative OD (rOD) values for comparison between plates [44,45]. The cutoff rOD was defined by the mean rOD value of negatives plus 12 standard deviations, which gave a confidence level of 99.9% from 4 negatives [55]. Each ELISA was performed twice (each in duplicate).

### Statistical analysis

Two-tailed Mann-Whitney test was used to determine the P values between two groups, the two-tailed Spearman correlation test the relationship between the rOD and rMFI values, and the receiver-operating characteristics (ROC) analysis the cutoffs of the rMFI and rOD ratios (GraphPad Prism 6). The 95% confidence interval (CI) was calculated by Excel.

## Results

### Multiplex NS1 IgG MIA can distinguish different primary flavivirus infections

**Fig 1:**
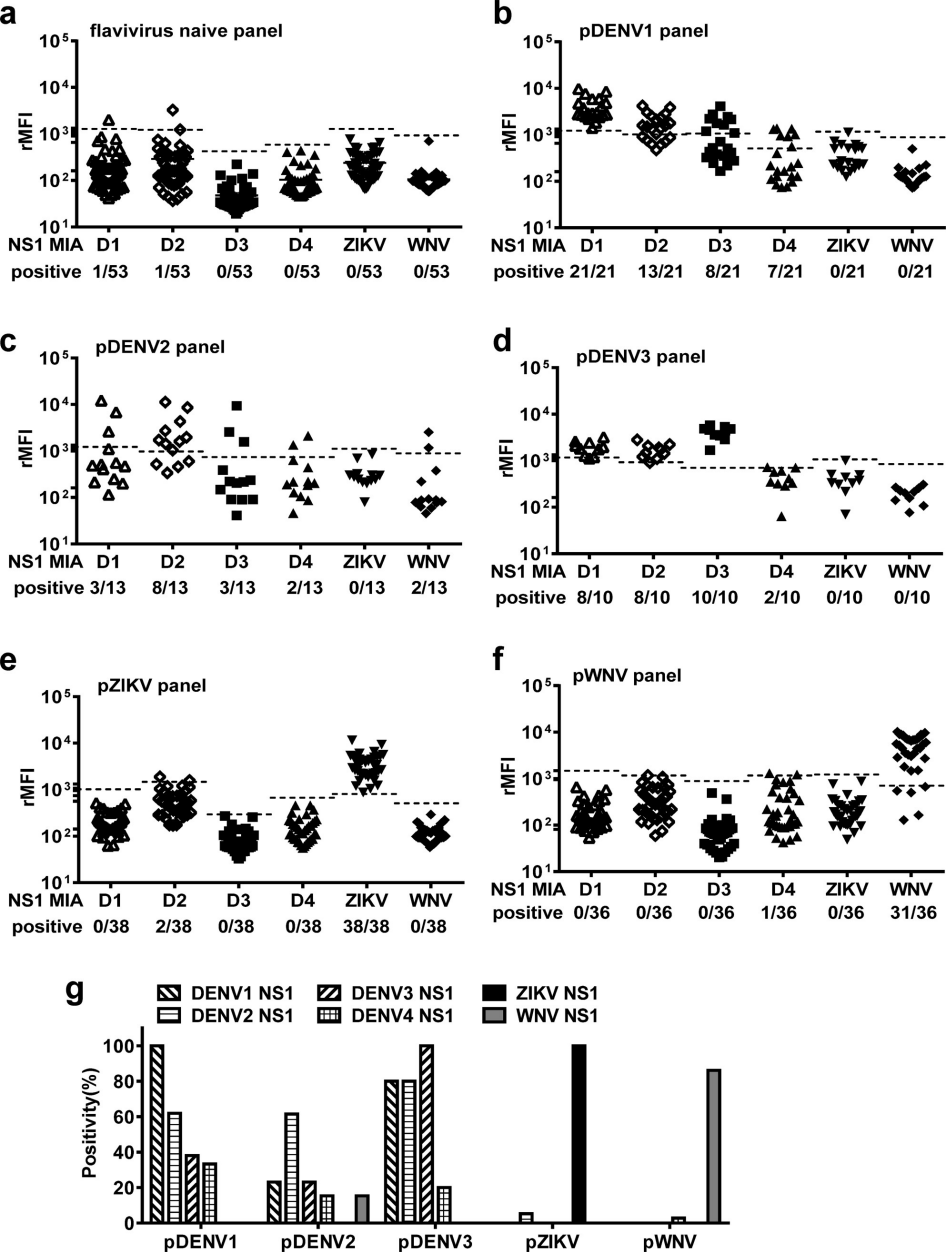
Results of the multiplex NS1 IgG MIA tested with different primary flavivirus infection and naïve panels. (a-f) Convalescent-phase and post-convalescent-phase samples of pDENV1 (b), pDENV2 (c), pDENV3 (d), pZIKV (e) and pWNV (f) panels as well as flavivirus naïve (a) panel were tested. (g) Detection rates of different NS1 IgG MIA by each panel. Data are the means of two separate experiments (each in duplicate). Dashed lines indicate cutoff rMFI; horizontal lines the means of each NS1 in panel a.

We first employed the multiplex NS1 IgG MIA to test samples from primary DENV (pDENV1, pDENV2 and pDENV3), pZIKV and pWNV infection panels. Compared with flavivirus-naïve panel, the pDENV1 panel recognized the NS1 proteins of DENV1 (100%) and other DENV serotypes (33.3 to 61.9%), but not those of different serocomplexes (ZIKV and WNV NS1 proteins) (Fig 1A and 1B). Similarly, the pDENV2 and pDENV3 panels recognized the NS1 protein of the homologous serotype (DENV2, DENV3) better than those of other serotypes (Fig 1C and 1D), but did not recognize ZIKV or WNV NS1 protein except two samples (recognizing WNV, 2/13). The pZIKV panel recognized ZIKV NS1 protein but not those of WNV and DENV except two sample recognizing DENV2 (2/38), whereas the pWNV panel recognized WNV proteins rather than those of ZIKV and DENV except one sample (recognizing DENV4, 1/36) (Fig 1E and 1F). Taken together, these findings suggested that primary infection panels recognized the homologous (infecting serotype) NS1 protein better than other NS proteins within the same serocomplex, and in general did not recognize an NS protein of different serocomplexes (Fig 1G).

### Cross-reactivity to multiple NS1 proteins after repeated flavivirus infections

**Fig 2:**
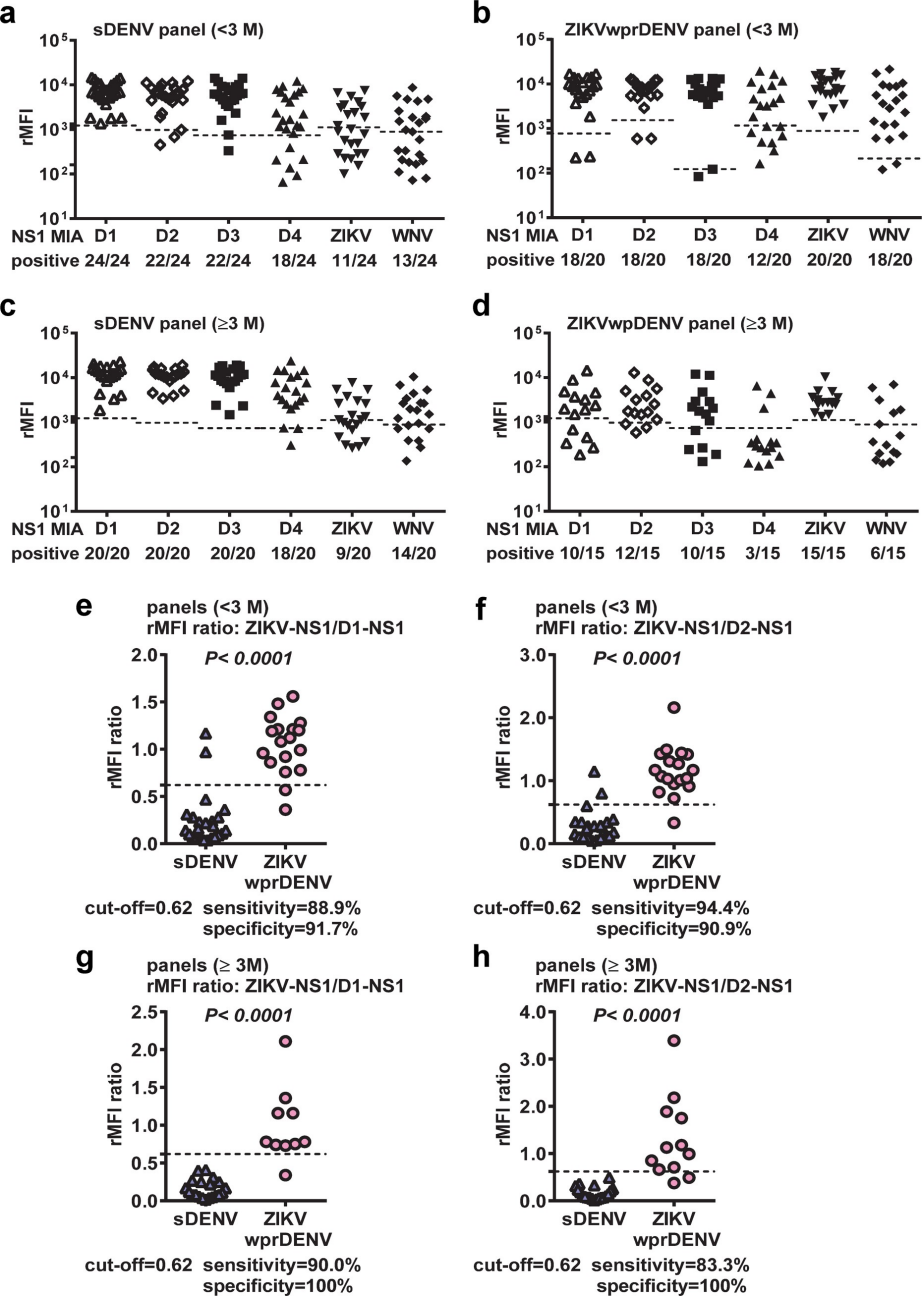
Results of the multiplex NS1 IgG MIA tested with repeated flavivirus infection panels. (a-d) These include sDENV panel at the convalescent (a) and post-convalescent (c) phases and ZIKVwprDENV panel at the convalescent (b) and post-convalescent (d) phases. Data are the means of two experiments (each in duplicate). Dashed lines indicate cutoff rMFI. (e-h) The rMFI ratio of ZIKV NS1 to DENV1 NS1 for the convalescent-phase (e) and post-convalescent-phase (g) samples, and the rMFI ratio of ZIKV NS1 to DENV2 NS1 for the convalescent-phase (f) and post-convalescent-phase (h) samples were shown. Data are the means of two experiments (each in duplicate). Dashed lines indicate cutoff rMFI ratio (0.62). The two-tailed Mann-Whitney test was used to compare two groups.

We next tested samples from sDENV and ZIKVwprDENV panels. For convalescent-phase samples, sDENV panel not only recognized NS1 proteins of DENV1-4 (66.7 to 100%) but also those of ZIKV and WNV (45.8 to 54.2%) (Fig 2A). The ZIKVwprDENV panel recognized ZIKV NS1 protein (100%) as well as DENV1-4 and WNV NS1 proteins (60.0 to 90.0%) (Fig 2B). A similar trend was observed for post-convalescent-phase samples (Fig 2C and 2D). These findings were in agreement with our previous reports based on NS1 IgG ELISAs [44,45], and suggested that after repeated flavivirus infections, such as sDENV and ZIKVwprDENV infections, anti-NS1 antibodies cross-reacted to multiple NS1 proteins, including those from prior exposure or sometimes those with no prior exposure.

### Distinction between sDENV and ZIKVwprDENV panels

Previously we reported that sDENV panel not only recognized DENV1 NS1 protein but also ZIKV NS1 protein in IgG ELISA (95.8 and 66.7%, respectively); similarly the ZIKVwprDENV panel recognized both ZIKV and DENV1 NS1 proteins (95.0 and 85.0%, respectively) [44]. Using the rOD ratio of ZIKV NS1 to DENV1 NS1 with a cutoff at 0.24, we can distinguish ZIKVwprDENV and sDENV panels. Since the same sDENV and ZIKVwprDENV panels recognized both DENV1 and ZIKV NS1 proteins in IgG MIA (Fig 2A and 2B), we calculated the ratio of relative median fluorescence intensity (rMFI) of ZIKV NS1 to that of DENV1 NS1 and found that a cutoff of the rMFI ratio at 0.62, as determined by ROC analysis, can distinguish these two panels with a sensitivity of 88.9% and specificity of 91.7% (Fig 2E). Since both panels also recognized DENV2 NS1 protein, we further calculated the ratio of rMFI of ZIKV NS1 to DENV2 NS1; interestingly a cutoff of the rMFI ratio at 0.62 was able to distinguish these two panels with a sensitivity of 94.4% and specificity of 90.9% (Fig 2F). Similar observations were found for post-convalescent-phase sDENV and ZIKVwprDENV panels; these two panels can be distinguished by a cutoff (0.62) of the rMFI ratio for ZIKV NS1 to DENV1 NS1 or DENV2 NS1 with a sensitivity/specificity of 90.0/100% or 83.3/100%, respectively (Fig 2G and 2H).

### Comparison between NS1 IgG MIA and ELISA

**Fig 3:**
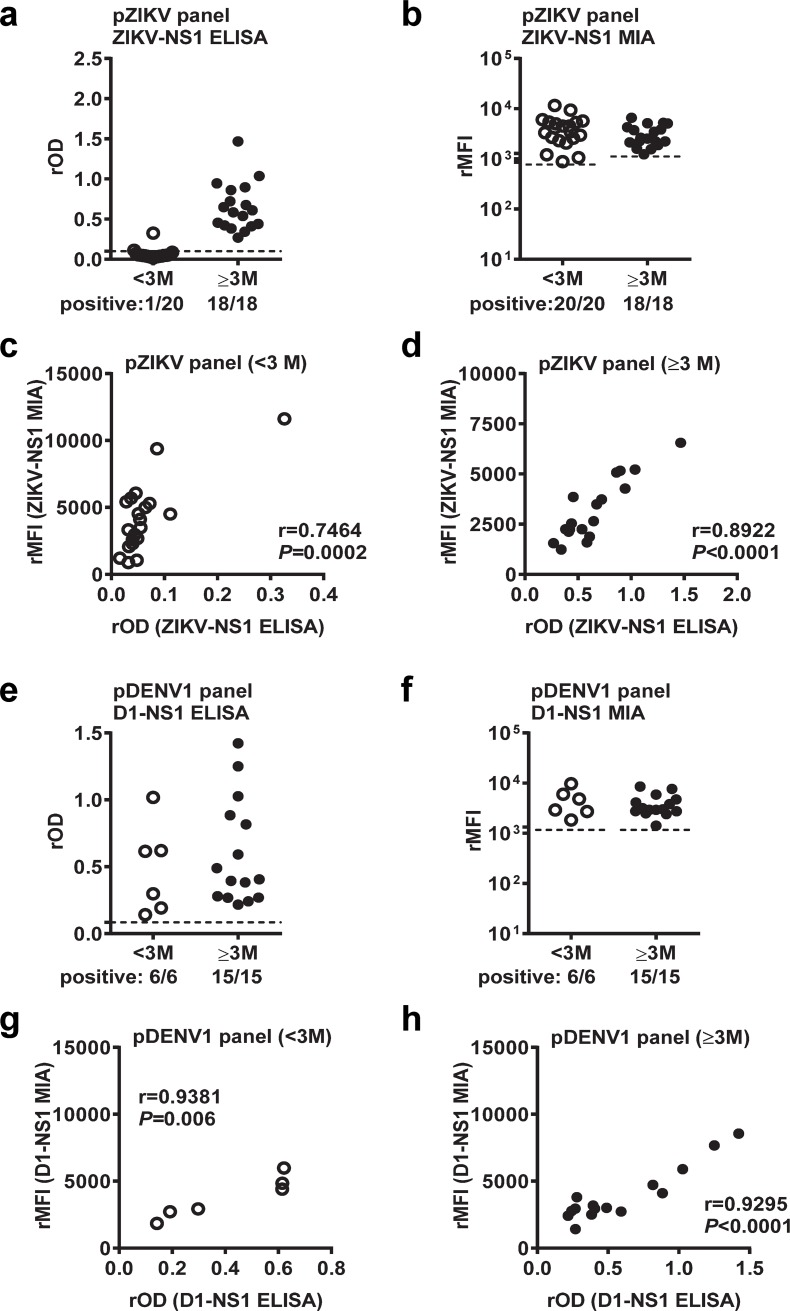
Comparison between NS1 IgG MIA and ELISA for pZIKV and pDENV1 panels. (a-d) The results of ZIKV NS1 IgG ELISA (a) and MIA (b) for convalescent- and post-convalescent-phase samples of pZIKV panel. Relationship between rOD and rMFI values for the convalescent-phase (c) and post-convalescent-phase (d) samples. (e-h) The results of DENV1 NS1 IgG ELISA (e) and MIA (f) for convalescent- and post-convalescent-phase samples of pDENV1 panel. Relationship between rOD and rMFI values for the convalescent-phase (g) and post-convalescent-phase (h) samples. Data are the means of two experiments (each in duplicate). Dashed lines indicate cutoff rOD or rMFI. The two-tailed Spearman correlation test was performed; r, correlation coefficient.

Since these panels have been tested with individual DENV1 to DENV4 and ZIKV NS1 IgG ELISAs previously [45], we compared the detection rates for each NS1 protein between ELISA and MIA. For the pZIKV panel, ZIKV NS1 ELISA had a detection rate of 100%, comparable to that of MIA, for the post-convalescent-phase samples, but only 5% for the convalescent-phase samples, which was much lower than that of MIA (100%) (Fig 3A and 3B). Although 19 convalescent-phase pZIKV samples were tested negative by ZIKV NS1 IgG ELISA, the relative optical density (rOD) values were positively correlated with the rMFI values (correlation coefficient r = 7464, *P* = 0.0002) (Fig 3C), suggesting that ZIKV NS1 MIA was more sensitive than ELISA. A positive correlation was also found between rOD and rMFI values for the post-convalescent-phase samples (r = 8922, *P*<0.0001) (Fig 3D). For pDENV1 panel, DENV1 NS1 ELISA and MIA had comparable detection rates (100%) for both convalescent and post-convalescent-phase samples (Fig 3E and 3F). Similarly, a positive correlation was found between rOD and rMFI values (Fig 3G and 3H).

**Fig 4:**
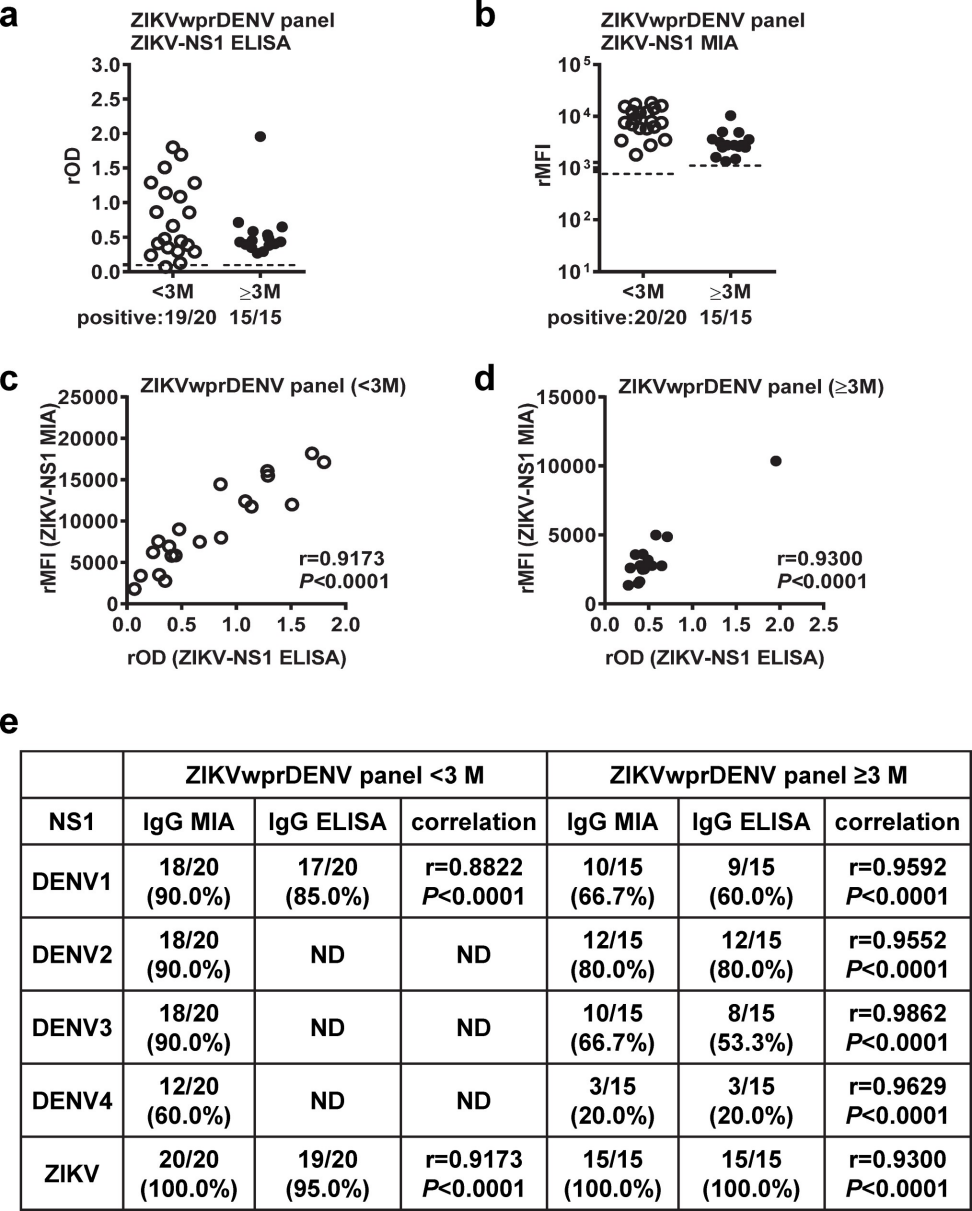
Comparison between NS1 IgG MIA and ELISA for ZIKVwprDENV panel. (a-d) The results of ZIKV NS1 IgG ELISA (a) and MIA (b) for convalescent- and post-convalescent-phase samples. Relationship between rOD and rMFI values for the convalescent-phase (c) and post-convalescent-phase (d) samples. Data are the means of two experiments (each in duplicate). Dashed lines indicate cutoff rOD or rMFI. The two-tailed Spearman correlation test was performed; r, correlation coefficient. (e) Summary of the relationship between rOD and rMFI values of different NS1 IgG ELISAs and MIAs for the convalescent-phase and post-convalescent-phase samples of ZIKVwprDENV panel.

For ZIKVwprDENV panels, ZIKV NS1 IgG ELISA and MIA had comparable detection rates for both convalescent and post-convalescent-phase samples (Fig 4A and 4B). A positive correlation was found between rOD and rMFI values for ZIKV NS1 as well as DENV1, DENV2, DENV3 and DENV4 NS1 tested (Fig 4C–4E). Similar observations were found for sDENV panels (S2 Fig).

### Sensitivity and specificity of the multiplex MIA

Table 1 summarizes the results of all samples tested with different NS1 proteins (DENV1, DENV2, DENV3, DENV4, DENV1, 2, 3 or 4, ZIKV and WNV) in the IgG MIA. For statistical analysis comparing different panels, one sample from each participant was included (S2 Table). The overall sensitivity of each DENV (DENV1, DENV2, DENV3) NS1 IgG MIA to detect different DENV infections ranged from 73.6 to 90.1% and specificity from 98.1 to 100% (Table 2). Interestingly, combination of four DENV NS1 IgG MIA increased the sensitivity to 94.5%, while maintaining the specificity of 97.2%, suggesting that this multiplex assay can be applied to detect DENV infections rather than distinguish different DENV serotypes. For the ZIKV NS1 IgG MIA, the overall sensitivity was 100% and specificity 87.9%. For the WNV NS1 IgG MIA, the overall sensitivity was 86.1% and specificity 78.4% (Table 2).

**Table 1:** Results of NS1 IgG MIA in different serum/plasma panels.

	No. of positive/total samples (%) in different serum/plasma panels ^a^							
NS1 IgG MIA	naïve	pWNV	pDENV1	pDENV2	pDENV3	pZIKV	sDENV	ZIKVwprDENV
DENV1	1/53(1.9%)	0/36(0%)	21/21(100%)	3/13 (23.1%)	8/10(80.0%)	0/38(0%)	44/44(100%)	28/35(80.0%)
DENV2	1/53(1.9%)	0/36(0%)	13/21(61.9%)	8/13 (61.5%)	8/10 (80.0%)	2/38(5.3%)	42/44(95.5%)	30/35(85.7%)
DENV3	0/53(0%)	0/36(0%)	8/21(38.1%)	3/13 (23.1%)	10/10(100%)	0/38(0%)	42/44(95.5%)	28/35(80.0%)
DENV4	0/53(0%)	1/36(2.8%)	7/21(33.3%)	2/13 (15.4%)	2/10(20.0%)	0/38(0%)	36/44(81.8%)	15/35(42.9%)
DENV1, 2, 3 or 4	1/53(1.9%)	1/36(2.8%)	21/21(100%)	8/13(61.5%)	10/10(100.0%)	2/38(5.3%)	44/44(100%)	30/35(85.7%)
ZIKV	0/53(0%)	0/36(0%)	0/21(0%)	0/13(0%)	0/10(0%)	38/38(100%)	20/44(45.5%)	35/35(100%)
WNV	0/53(0%)	31/36(86.1%)	0/21(0%)	2/13(15.4%)	0/10(0%)	0/38(0%)	27/44(61.4%)	24/35(68.6%)

^a^ MIA: microsphere immunoassay; pWNV: primary WNV infection; pDENV1: primary DENV1 infection; pDENV2: primary DENV2 infection; pDENV3: primary DENV3 infection; pZIKV: primary ZIKV infection; sDENV: secondary DENV infection; ZIKVwprDENV: ZIKV infection with previous DENV infection.

**Table 2:** Sensitivity and specificity of different NS1 IgG MIA ^a^.

	DENV1 NS1		DENV2 NS1		DENV3 NS1		DENV1, 2, 3 or 4 NS1		ZIKV NS1		WNV NS1	
Panels ^b^	% Sens(95% CI)	% Spec(95% CI)	% Sens(95% CI)	% Spec(95% CI)	% Sens(95% CI)	% Spec(95% CI)	% Sens(95% CI)	% Spec(95% CI)	% Sens(95% CI)	% Spec(95% CI)	% Sens(95% CI)	% Spec(95% CI)
overall	89.7(83.3–92.9)	99.1(97.2–100)	83.9(76.2–87.9)	98.1(95.6–99.4)	73.6(64.3–78.3)	100(100–100)	94.3(89.4–96.8)	97.2(94.1–98.8)	100(100–100)	87.9(82.9–90.4)	86.1(74.8–91.9)	78.4(72.1–81.6)
pDENV1	100(100–100)	NA	58.8(35.4–70.7)	NA	29.4(7.8–40.5)	NA	100(100–100)	NA	NA	100(100–100)	NA	100(100–100)
pDENV2	42.9(6.2–61.6)	NA	71.4(38.0–88.5)	NA	42.9(6.2–61.6)	NA	71.4(38.0–88.5)	NA	NA	100(100–100)	NA	71.4(38.0–88.5)
pDENV3	100(100–100)	NA	100(100–100)	NA	100(100–100)	NA	100(100–100)	NA	NA	100(100–100)	NA	100(100–100)
sDENV	100(100–100)	NA	95.5(89.3–98.6)	NA	95.5(89.3–98.6)	NA	100(100–100)	NA	NA	54.6(39.8–62.1)	NA	38.6(24.3–46.0)
Naïve	NA	98.1(94.5–100)	NA	98.1(94.5–100)	NA	100(100–100)	NA	98.1(94.5–100)	NA	100(100–100)	NA	100(100–100)
pWNV	NA	100(100–100)	NA	100(100–100)	NA	100(100–100)	NA	97.2(91.9–100)	NA	100(100–100)	86.1(74.8–91.9)	NA
pZIKV ^c^	NA	100(100–100)	NA	94.4(83.9–99.8)	NA	100(100–100)	NA	94.4(83.9–99.8)	10(100–100)	NA	NA	100(100–100)
ZIKVwprDENV ^c^	NA	33.3(9.5–45.5)	NA	20(0–30.3)	NA	33.3(9.5–45.5)	NA	20(0–30.3)	100(100–100)	NA	NA	60.0(35.2–72.7)

^a^ MIA: microsphere immunoassay; Sens: sensitivity. Spec: specificity; CI: confidence interval. For those with repeated samples, only one sample from each subject was included.

^b^ pDENV1: primary DENV1 infection; pDENV2: primary DENV2 infection; pDENV3: primary DENV3 infection; pWNV: primary WNV infection; pZIKV: primary ZIKV infection; sDENV: secondary DENV infection; ZIKVwprDENV: ZIKV infection with previous DENV infection.

^c^ For pZIKV and ZIKVwprDENV panels, samples at post-convalescent phase (≥3 months post-symptom onset) were presented.

## Discussion

**Fig 5:**
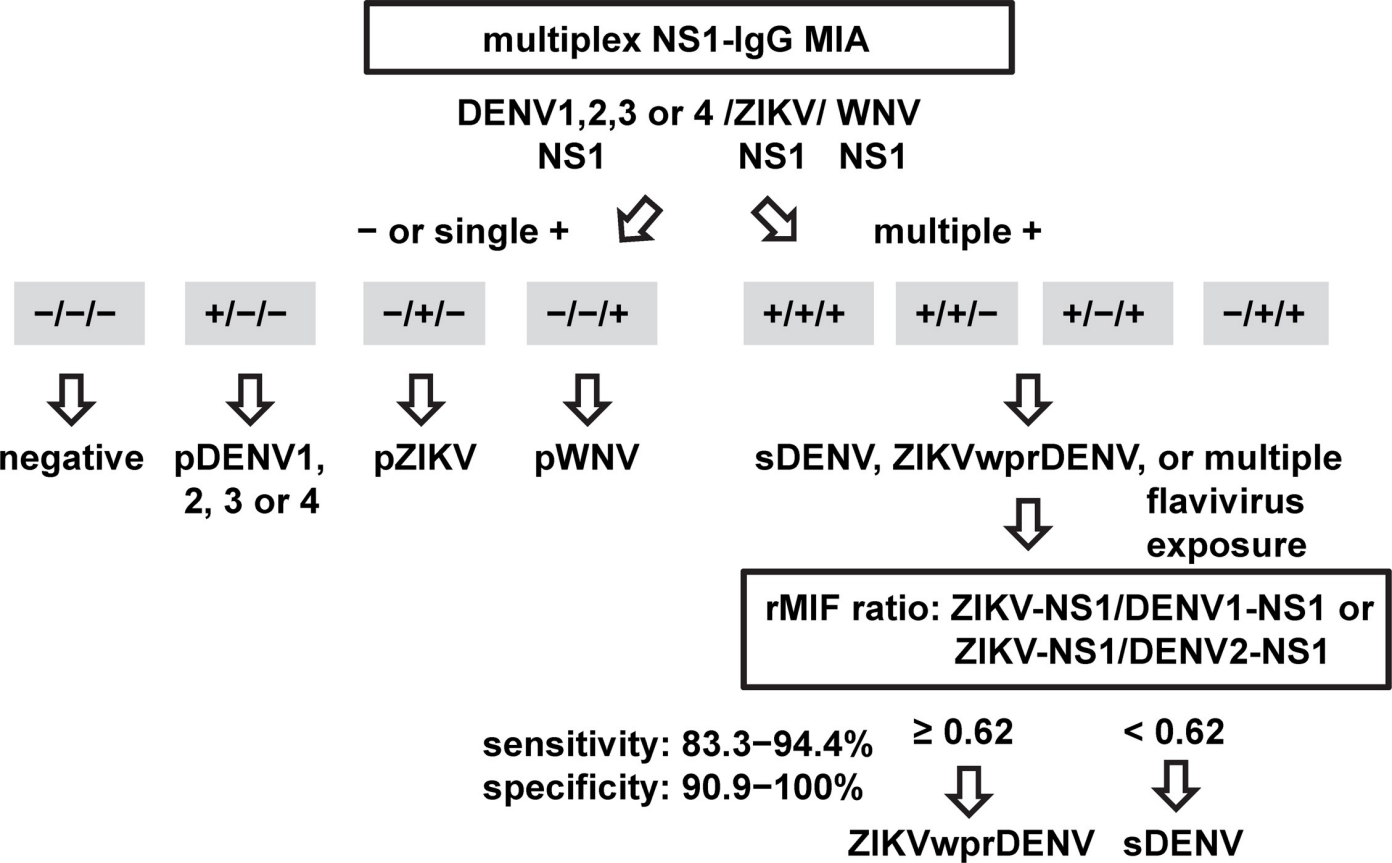
Proposed algorithm of using multiplex NS1 IgG MIA to distinguish three flavivirus infections. Based on positivity to NS1 proteins of three serocomplexes (DENV [DENV1, 2, 3 or 4], ZIKV and WNV NS1), the samples that were negative to all NS1 proteins or positive to NS1 protein of one serocomplex (DENV, ZIKV or WNV) could be flavivirus naïve or primary DENV, pZIKV or pWNV infection. For samples that were positive to NS1 proteins of two or more serocomplexes, the rMFI ratio of ZIKV NS1 to DENV1 or DENV2 NS1 was calculated to distinguish sDENV and ZIKVwprDENV infections.

In this study, we developed a high-throughput and multiplex IgG MIA using NS1 proteins of DENV1 to DENV4, ZIKV and WNV to detect and distinguish various DENV, ZIKV and WNV infections. Based on the results, we propose an algorithm to discriminate primary DENV, pZIKV and pWNV infections, sDENV infection and ZIKVwprDENV infection (Fig 5). Previous studies of flavivirus serodiagnosis mainly focused on two flaviviruses. Compared with a recent study of IgG MIA containing ZIKV and DENV antigens, our multiplex IgG MIA consists of 6 antigens (DENV1 to DENV4, WNV and ZIKV NS1 proteins) plus two controls (BSA and PBS) [56]. To our knowledge, this is the first report of a single serological test to detect three flavivirus infections. Our findings that combination of DENV1 to DENV4 NS1 IgG MIA increased the sensitivity to 94.3% while maintaining a specificity of 97.2% and that the rMFI ratio of ZIKV NS1 to DENV1 or DENV2 NS1 can distinguish ZIKVwprDENV and sDENV infections with a sensitivity of 83.3–94.4% and specificity of 90.9–100.0% have important applications to serodiagnosis and serosurveillance of DENV and ZIKV infections in regions where both viruses co-circulate.

Generally in agreement with our recent study of individual DENV NS1 ELISAs [45], we found that DENV1 and DENV3 NS1 IgG MIAs can detect primary DENV infection of the homologous serotype with a sensitivity (100%) higher than that for heterologous serotypes (25.0 to 100%) (Table 2). DENV1, DENV2 and DENV3 NS1 IgG MIAs can detect secondary DENV infection with a sensitivity of 95.5 to 100%. This was also consistent with our previous study using Western blot analysis, in which anti-NS1 antibodies recognized NS1 protein predominantly of the infecting serotype after primary DENV infection and multiple NS1 proteins after secondary infection [13]. Taken together, due to the variable and extensive cross-reactivity of anti-NS1 antibodies after primary and secondary DENV infections, respectively, it is difficult to use a single NS1 IgG MIA or ELISA to identify the infecting DENV serotype. Notably, the combination of four DENV NS1 IgG MIA can detect different primary and secondary DENV infections with a sensitivity of 94.3% and specificity of 97.2% (Table 2), suggesting the feasibility and application of this multiplex NS1 IgG MIA to detect DENV infection rather than distinguish DENV serotypes.

The overall sensitivity of the ZIKV NS1 IgG MIA was 100% and the specificity was 87.9%, primarily due to the cross-reactivity of the sDENV panel (Table 2). The sensitivity (100%) was higher than or comparable with those previously reported (79 to 100%) using the Euroimmun ZIKV NS1 IgG ELISA kit [34–37]. The ZIKV NS1 blockade of binding ELISA had an overall specificity of 91.4–92.6%, which reduced to 77.6–90.5% when comparing with sDENV panel [38,39]. A recently reported ZIKV NS1 IgG3 ELISA had a sensitivity of 97% based on samples from Salvador, but it reduced to 83% when comparing with samples outside of Salvador [32]. A previous study of multiplex IgG MIA including ZIKV NS1 reported a sensitivity of 100% and specificity of 78% for pZIKV panel based on PRNT results, however, the sDENV and ZIKVwprDENV panels were not distinguished [56]. For the WNV NS1 IgG MIA, the overall sensitivity was 86.1% probably due to sampling during the early convalescent-phase for this pWNV panel (S1 Table), and the specificity was 78.4%, mainly due to the cross-reactivity from the sDENV and ZIKVwprDENV panels (Table 2). Using the rMFI ratio of ZIKV NS1 to DENV1 or DENV2 NS1, we can distinguish ZIKVwprDENV and sDENV panels with a sensitivity of 83.3–94.4% and specificity of 90.9–100.0%. This was consistent with our previous reports of IgG ELISAs using the rOD ratio of ZIKV NS1 to DENV1 NS1 or mixed DENV1-4 NS1 to distinguish these two panels with a sensitivity of 91.7–94.1% and specificity of 87.0–95.0% [44,45]. It is worth noting since DENV3 and DNV4 NS1 proteins were not recognized by several samples from the sDENV and ZIKVwprDENV panels (Fig 2A and 2D), they were not included in the analysis of the rMFI ratio.

Comparing the results of individual NS1 IgG MIA in this study and those of NS1 IgG ELISA reported previously [45], we found comparable detection rates between MIA and ELISA, and positive correlations between the rMFI and rOD values for both convalescent-phase and post-convalescent-phase samples of most panels tested including pDENV1, sDENV and ZIKVwprDENV panels except pZIKV panel (Figs 3 and 4 and S2 Fig). Of note, the IgG MIA detection rates for DENV1-4 for the post-convalescent-phase ZIKVwprDENV panel were much lower than those for the sDENV panel (Fig 4E and S2E Fig), suggesting that prior DENV exposure of the ZIKVwprDENV panel may have been only to a single DENV serotype. For the convalescent-phase pZIKV panel, the higher detection rate of ZIKV NS1 IgG MIA (100%) than that of ELISA (5%) and the positive correlation between rOD and rMFI values suggest that MIA was more sensitive than ELISA (Fig 3A–3C). Thus, we did not observe a trend of increased detection rates of NS1 IgG MIA from convalescent to post-convalescent phases for primary infection panels (pZIKV, pDENV1) (Fig 3B and 3F) as previously reported for NS1 IgG ELISA and blockade of binding of NS1 ELISA [38,45]. Notably we incubated 16 ng antigen coated on each well with 50 μL of serum (1:400) in ELISA, whereas we incubated ~10 ng antigen (in 1250 beads) with serum (final dilution 1:200) per well in MIA. The higher concentration of serum and more surface area of antigen coupled on beads may account for the higher sensitivity of the IgG MIA compared with IgG ELISA for the pZIKV convalescent-phase panel.

Although neutralization tests are still considered a confirmatory assay, they are time-consuming and can be performed only in reference laboratories. Compared with PRNT and ELISA, the multiplex MIA requires less time (2.5 h vs. 7 h for ELISA and 5–6 days for PRNT) and less sample volume (1 μL vs. 8 μL for ELISA and 144 μL for PRNT for 8 antigens or viruses). The newly developed multiplex NS1 IgG MIA could have wide-ranging applications, such as serodiagnosis, blood screening, serosurveillance of ZIKV, DENV and WNV infections, and retrospective study of ZIKV infection among pregnant women with CZS [57,58]. The current octaplex (6 NS1 antigens plus PBS and BSA controls) IgG MIA serves as a “proof-of-concept” assay to demonstrate that NS1-based MIA can distinguish three flavivirus infections; incorporation of other antigens would increase the detection capacity for different clinical settings and studies. These together would further our understanding of the epidemiology, pathogenesis and complications of ZIKV in regions where multiple flaviviruses co-circulate [1–4].

There are several limitations of this study. First, due to limited samples of < 3 months PSO from patients with primary DENV infection (S1 Table), the study focused on NS1 IgG MIA. Future studies on NS1-based IgM MIA are warranted. Second, despite the availability of two-time point samples for the pZIKV and ZIKVwprDENV panels, future studies involving more sequential samples are needed to validate these observations. Additionally, the sample size in each panel with well-documented infection is small. Third, although this multiplex assay can distinguish various panels of samples with three flavivirus infections, future tests that can distinguish other pathogenic flaviviruses such as JEV, YFV and tick-borne encephalitis virus (TBEV) remain to be exploited [59,60]. Moreover, samples with well-documented repeated flavivirus infections such as DENV with previous ZIKV infection and sequential DENV and WNV infections are lacking and remain to be investigated in the future. In light of the successful implementation of several flavivirus vaccines and vaccine trials in flavivirus-endemic regions, serological tests that can distinguish ZIKV infection from vaccinations with DENV, JEV, YFV and TBEV vaccines are warranted [59,60].

## Supporting information

S1 Fig Determination of rMFI and cutoff rMFI. (a) Positive control (PC), (b) flavivirus naïve, and (c) pDENV1 panels. The MFI values for each NS1 antigen were divided by the mean MFI value of one positive control (MFI~10^4^) and multiplied by 10^4^ to calculate to rMFI. The cutoff rMFI for each NS1 antigen was defined by the mean rMFI value of 19 flavivirus-naïve samples plus 5 standard deviations, Data are the means of two experiments (each in duplicate). Dashed lines indicate cutoff rMFI; horizontal lines the means of each NS1 in panels a and b.
(TIF)

S2 Fig Comparison between NS1 IgG MIA and ELISA for sDENV panel. (a-d) The results of ZIKV NS1 IgG ELISA (a) and MIA (b) for convalescent- and post-convalescent-phase samples. Relationship between rOD and rMFI values for convalescent-phase (c) and post-convalescent-phase (d) samples. Data are the means of two experiments (each in duplicate). Dashed lines indicate cutoff rOD or rMFI. The two-tailed Spearman correlation test was performed; r, correlation coefficient. (e) Relationship between rOD and rMFI values of DENV1-4 NS1 IgG ELISA and MIA for convalescent-phase and post-convalescent-phase samples.
(TIFF)

S1 Table Sampling time, serotypes and sources of different serum/plasma panels. (DOCX)

S2 Table Results of NS1 IgG MIA in different serum/plasma panels. (DOCX)
