# Supplementary figures and images for: A high-throughput and multiplex microsphere immunoassay based on non-structural protein 1 can discriminate three flavivirus infections
Source: PLoS Negl Trop Dis. 2019 Aug 23;13(8):e0007649. doi: 10.1371/journal.pntd.0007649 (PMC6707547; doi:10.1371/journal.pntd.0007649)

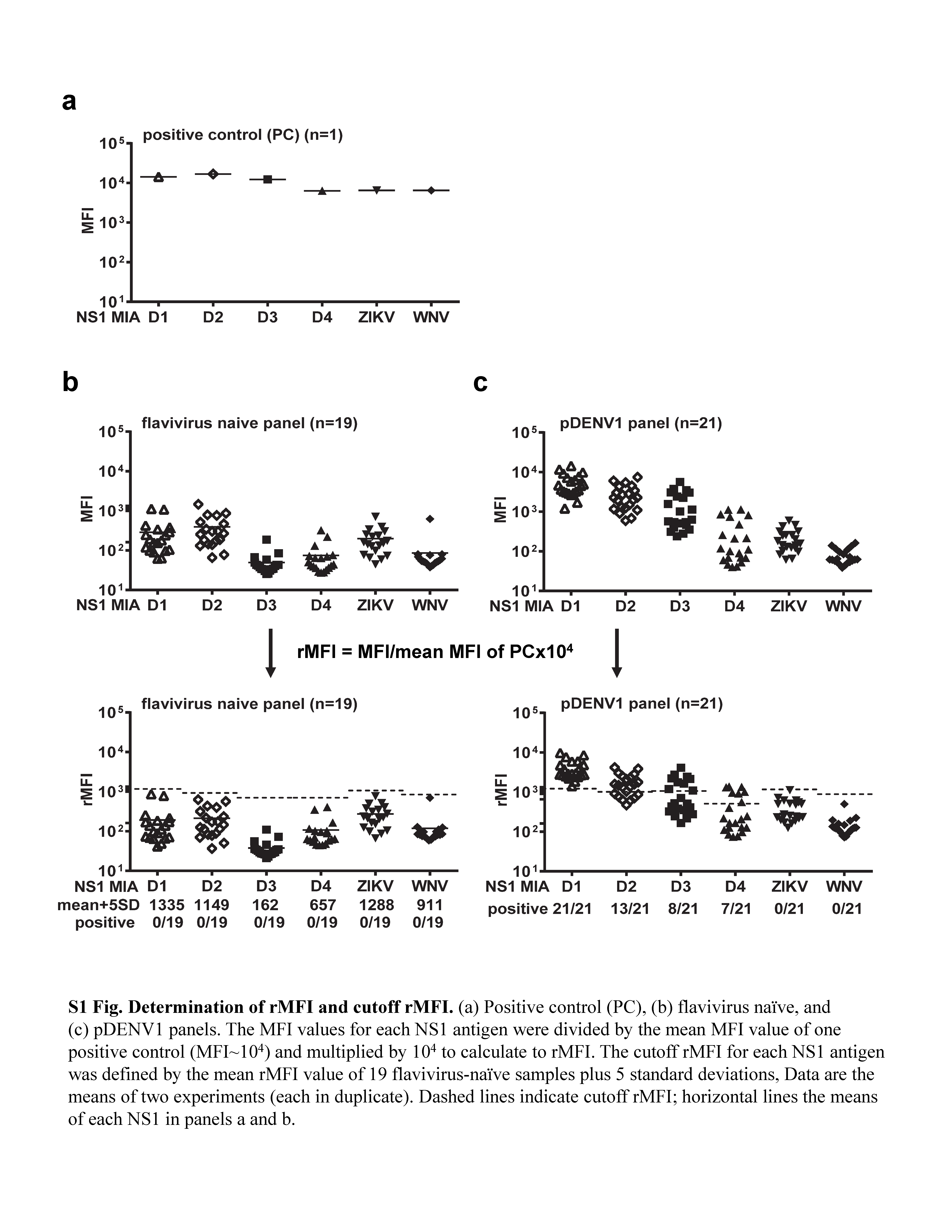

Supplement: S1 Fig — (a) Positive control (PC), (b) flavivirus naïve, and (c) pDENV1 panels. The MFI values for each NS1 antigen were divided by the mean MFI value of one positive control (MFI~104) and multiplied by 104 to calculate to rMFI. The cutoff rMFI for each NS1 antigen was defined by the mean rMFI value of 19 flavivirus-naïve samples plus 5 standard deviations, Data are the means of two experiments (each in duplicate). Dashed lines indicate cutoff rMFI; horizontal lines the means of each NS1 in panels a and b. (TIF) [file pntd.0007649.s001.tif]

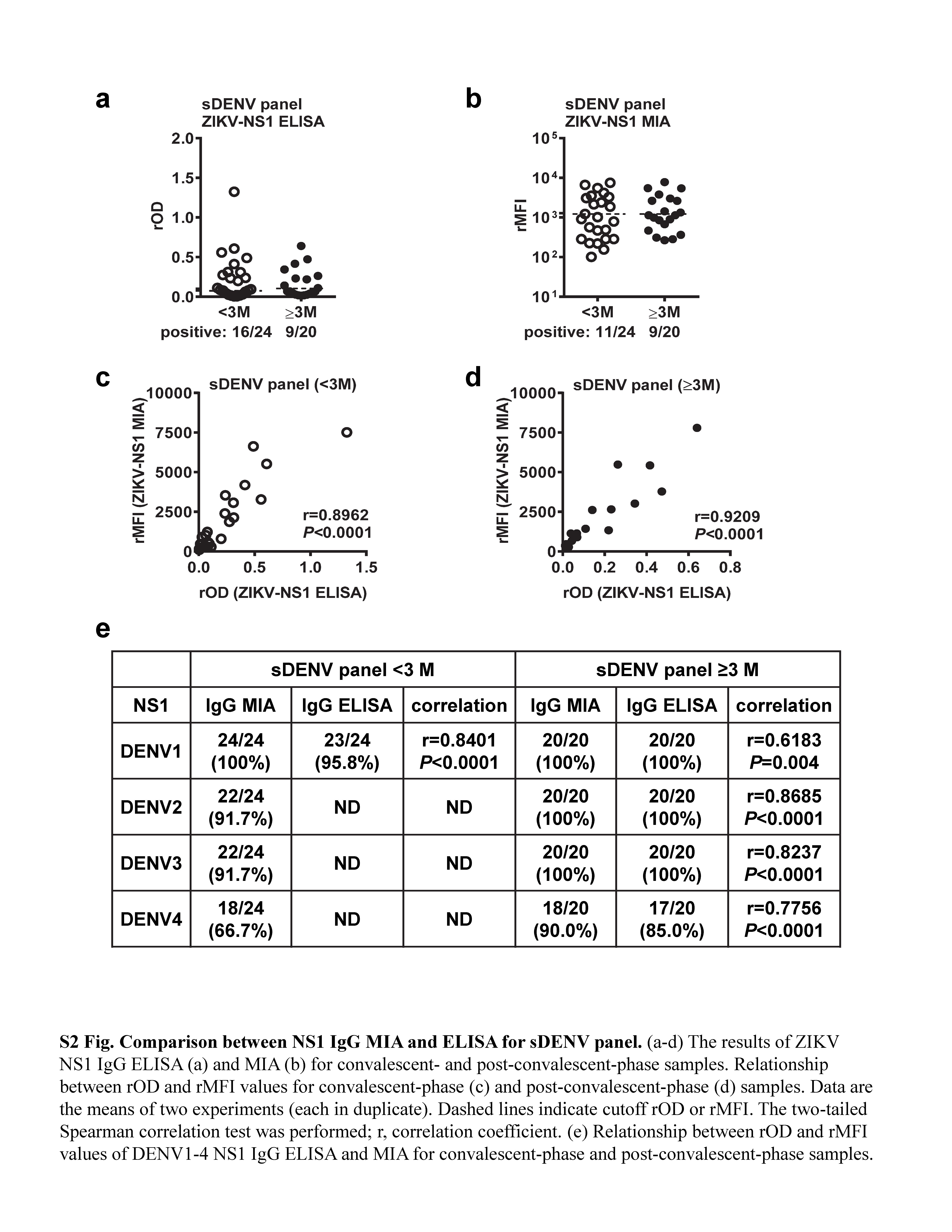

Supplement: S2 Fig — (a-d) The results of ZIKV NS1 IgG ELISA (a) and MIA (b) for convalescent- and post-convalescent-phase samples. Relationship between rOD and rMFI values for convalescent-phase (c) and post-convalescent-phase (d) samples. Data are the means of two experiments (each in duplicate). Dashed lines indicate cutoff rOD or rMFI. The two-tailed Spearman correlation test was performed; r, correlation coefficient. (e) Relationship between rOD and rMFI values of DENV1-4 NS1 IgG ELISA and MIA for convalescent-phase and post-convalescent-phase samples. (TIFF) [file pntd.0007649.s002.tiff]
