# Supplementary material for: A high-throughput and multiplex microsphere immunoassay based on non-structural protein 1 can discriminate three flavivirus infections
Source: PLoS Negl Trop Dis. 2019 Aug 23;13(8):e0007649. doi: 10.1371/journal.pntd.0007649 (PMC6707547; doi:10.1371/journal.pntd.0007649)
Supplement: S1 Table — (DOCX) [file pntd.0007649.s003.docx]

**S1 Table. Sampling time, serotypes and sources of different serum/plasma panels**

| Panel ^a^ | No. of subjects /samples | Category (No. of subjects/samples) | Sampling time PSO ^b^ mean and [range] | Sources (No.) of samples and year |
| --- | --- | --- | --- | --- |
| pDENV1 | 17/21 | convalescent (2/6) | 47 [19−89] days | Taiwan (6), 2006-9 Hawaii (9), 2015 Nicaragua (6), 2006-8 |
|  |  | post-convalescent (15/15) | 6.8 [3−15] months |  |
| pDENV2 | 7/13 | post-convalescent (7/13) | 24 [3−96] months | Taiwan (5), 2006-9 Nicaragua (8), 2006-8 |
| pDENV3 | 4/10 | post-convalescent (4/10) | 11 [3−19] months | Taiwan (2), 2006-9 Nicaragua (8), 2006-8 |
| pWNV | 36/36 | early convalescent (36/36) | not applicable ^c^ | U.S. ARC, 2006-15 |
| pZIKV | 20/38 ^d^ | convalescent (20/20) | 17 [14−24] days | Nicaragua, 2016 |
|  |  | post-convalescent (18/18) | 6.9 [6−8] months |  |
| ZIKVwprDENV | 20/35 ^d^ | convalescent (20/20) | 16 [14−19] days | Nicaragua, 2016 |
|  |  | post-convalescent (15/15) | 7.0 [6−8] months |  |
| sDENV | 44/44 | convalescent (24/24) | 14 [8−35] days | Taiwan, 2001-2 |
|  |  | post-convalescent (20/20) | 9.4 [3−12] months | Taiwan (2), 2006-9 Nicaragua (18), 2006-8 |
| flavivirus-naïve | 53/53 | seroprevalence study | not applicable | Taiwan (53), 2015-16 |
| ^a^ pDENV1: primary DENV1 infection; pDENV2: primary DENV2 infection; pDENV3: primary DENV3 infection; pWNV: primary WNV infection; pZIKV: primary ZIKV infection; sDENV: secondary DENV infection; ZIKVwprDENV: ZIKV infection with previous DENV infection. ^b^ PSO: post-symptom onset. ^c^ Index samples tested positive for WNV transcription-mediated amplification, IgM and IgG from blood donors at the American Red Cross (ARC). ^d^ 20 subjects from each panel provided convalescent samples and some of them provided post-convalescent samples. | | | | |
