# Supplementary material for: A high-throughput and multiplex microsphere immunoassay based on non-structural protein 1 can discriminate three flavivirus infections
Source: PLoS Negl Trop Dis. 2019 Aug 23;13(8):e0007649. doi: 10.1371/journal.pntd.0007649 (PMC6707547; doi:10.1371/journal.pntd.0007649)
Supplement: S2 Table — (DOCX) [file pntd.0007649.s004.docx]

**S2 Table. Results of NS1 IgG MIA in different serum/plasma panels**

|  | No. of positive/total samples (%) in different serum/plasma panels ^a^ | | | | | | | |
| --- | --- | --- | --- | --- | --- | --- | --- | --- |
| NS1 IgG MIA | naïve | pWNV | pDENV1 | pDENV2 | pDENV3 | pZIKV ^b^ | sDENV | ZIKV wprDENV^b^ |
| DENV1 | 1/53 (1.9%) | 0/36 (0%) | 17/17 (100%) | 3/7 (42.9.0%) | 4/4 (100%) | 0/18 (0%) | 44/44 (100%) | 10/15 (66.7%) |
| DENV2 | 1/53 (1.9%) | 0/36 (0%) | 10/17 (58.8%) | 5/7 (71.4%) | 4/4 (100%) | 1/18 (5.6%) | 42/44 (95.5%) | 12/15 (80.0%) |
| DENV3 | 0/53 (0%) | 0/36 (0%) | 5/17 (29.4%) | 3/7 (42.9.0%) | 4/4 (100%) | 0/18 (0%) | 42/44 (95.5%) | 10/15 (66.7%) |
| DENV4 | 0/53 (0%) | 1/36 (2.8%) | 4/17 (23.5%) | 2/7 (28.6%) | 2/4 (50.0%) | 0/18 (0%) | 36/44 (81.8%) | 3/15 (20.0%) |
| DENV1, 2, 3 or 4 | 1/53 (1.9%) | 1/36 (2.8%) | 17/17 (100%) | 5/7 (71.4%) | 4/4 (100%) | 1/18 (5.6%) | 44/44 (100%) | 12/15 (80.0%) |
| ZIKV | 0/53 (0%) | 0/36 (0%) | 0/17 (0%) | 0/7 (0%) | 0/4 (0%) | 18/18 (100%) | 20/44 (45.5%) | 15/15 (100%) |
| WNV | 0/53 (0%) | 31/36 (86.1%) | 0/17 (0%) | 2/7 (28.6%) | 0/4 (0%) | 0/18 (0%) | 27/44 (61.4%) | 6/15 (40.0%) |
| ^a^ MIA: microsphere immunoassay; pWNV: primary WNV infection; pDENV1: primary DENV1 infection; pDENV2: primary DENV2 infection; pDENV3: primary DENV3 infection; pZIKV: primary ZIKV infection; sDENV: secondary DENV infection; ZIKVwprDENV: ZIKV infection with previous DENV infection. ^b^ For those with repeated samples, only one sample from each subject was included. For pZIKV and ZIKVwpDENV panels, samples at post-convalescent phase (≥3 months post-symptom onset) were presented. | | | | | | | | |
